# Supplementary material for: LETR1 is a lymphatic endothelial-specific lncRNA governing cell proliferation and migration through KLF4 and SEMA3C
Source: Nat Commun. 2021 Feb 10;12:925. doi: 10.1038/s41467-021-21217-0 (PMC7876020; doi:10.1038/s41467-021-21217-0)
Supplement: Supplementary file 1 — Supplementary Information [file 41467_2021_21217_MOESM1_ESM.pdf]

SUPPLEMENTARY INFORMATION

LETR1 is a lymphatic endothelial-specific lncRNA governing cell proliferation and migration through KLF4 and SEMA3C

Luca Ducoli, Saumya Agrawal, Eliane Sibler, Tsukasa Kouno, Carlotta Tacconi, Chung-Chao Hon, Simone D. Berger, Daniela Müllhaupt, Yuliang He, Jihye Kim, Marco D'Addio, Lothar C. Dieterich, Piero Carninci, Michiel J. L. de Hoon, Jay W. Shin and Michael Detmar

Supplementary Figure 1

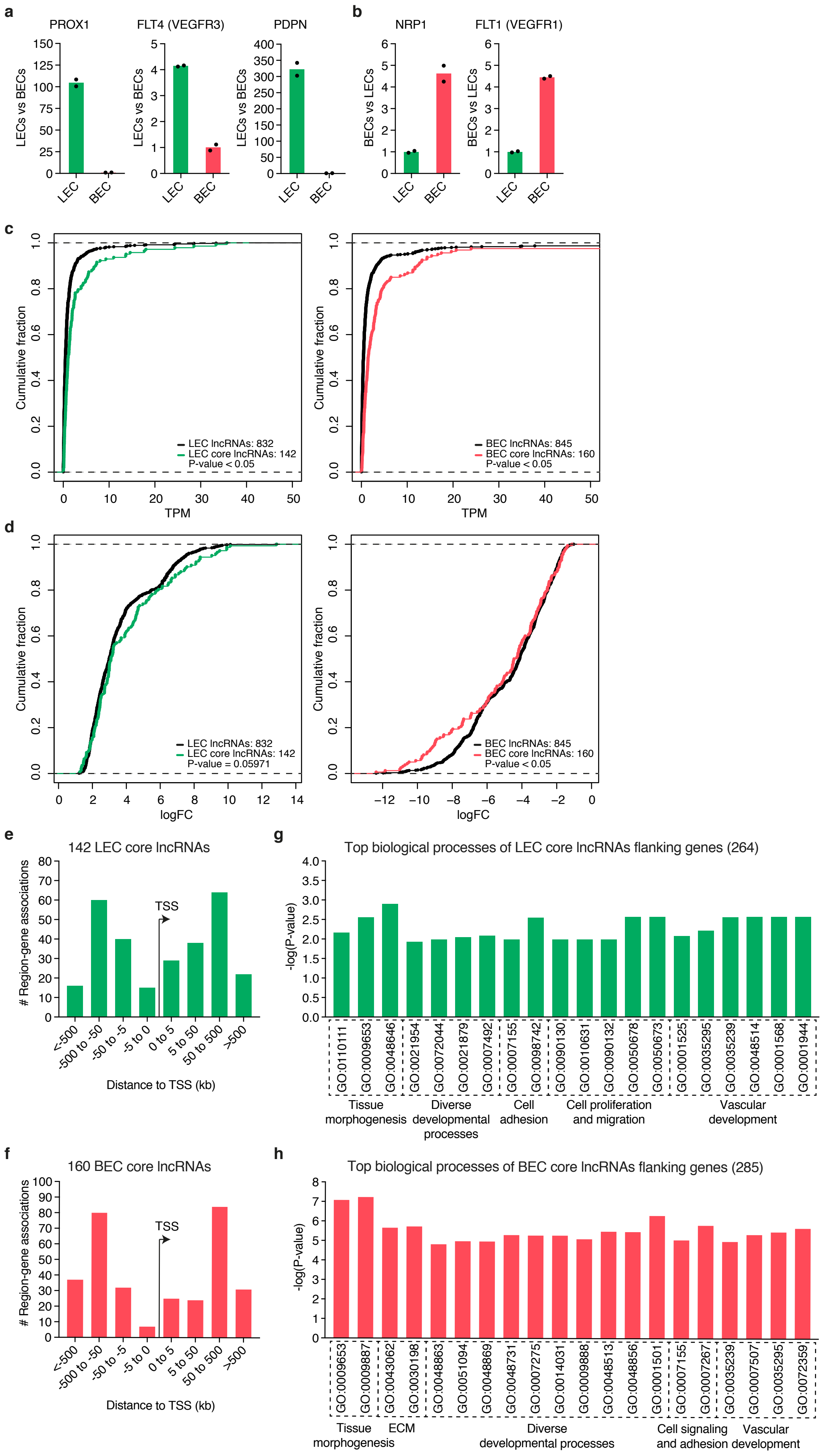

Supplementary Figure 1: Confirmation of LEC and BEC identity and analysis of BEC and LEC core lncRNA flanking protein-coding genes.

(a, b) Validation of LEC and BEC identities through expression analysis of LEC (a, PROX-1, FLT4, PDPN) and BEC (b, NRP1, FLT1) specific markers using qPCR. Bars represent fold change (FC) to either average LEC or BEC expression as mean values (n = 2). RPLP0 was used as the housekeeping gene.

(c, d) Cumulative fraction analysis of expression (c) and $\log_2$ fold change (log2FC) (d) distributions of LEC- and BEC-associated lncRNA detected by RNA-seq (832 and 845, respectively) and LEC and BEC core lncRNA datasets (142 and 160, respectively). P-values were calculated using the two-sample Kolmogorov-Smirnov test.

(e, f) Distribution of flanking genes determined with GREAT[1] of LEC (e) and BEC (f) core lncRNAs by the use of the association rule "two nearest genes" with a maximal extension from the lncRNA transcriptional start sites (TSS) of 10 Mb.

(g, h) Top significant (P-value < 0.05) enriched biological processes of LEC (g) and BEC (h) core lncRNA flanking genes, using gProfileR package[2] (relative depth 2-5). Terms were manually ordered according to their related biological meaning. Only genes with expression values (TPM & CPM) > 0.5 in LECs or BECs were used as background.

Supplementary Figure 2

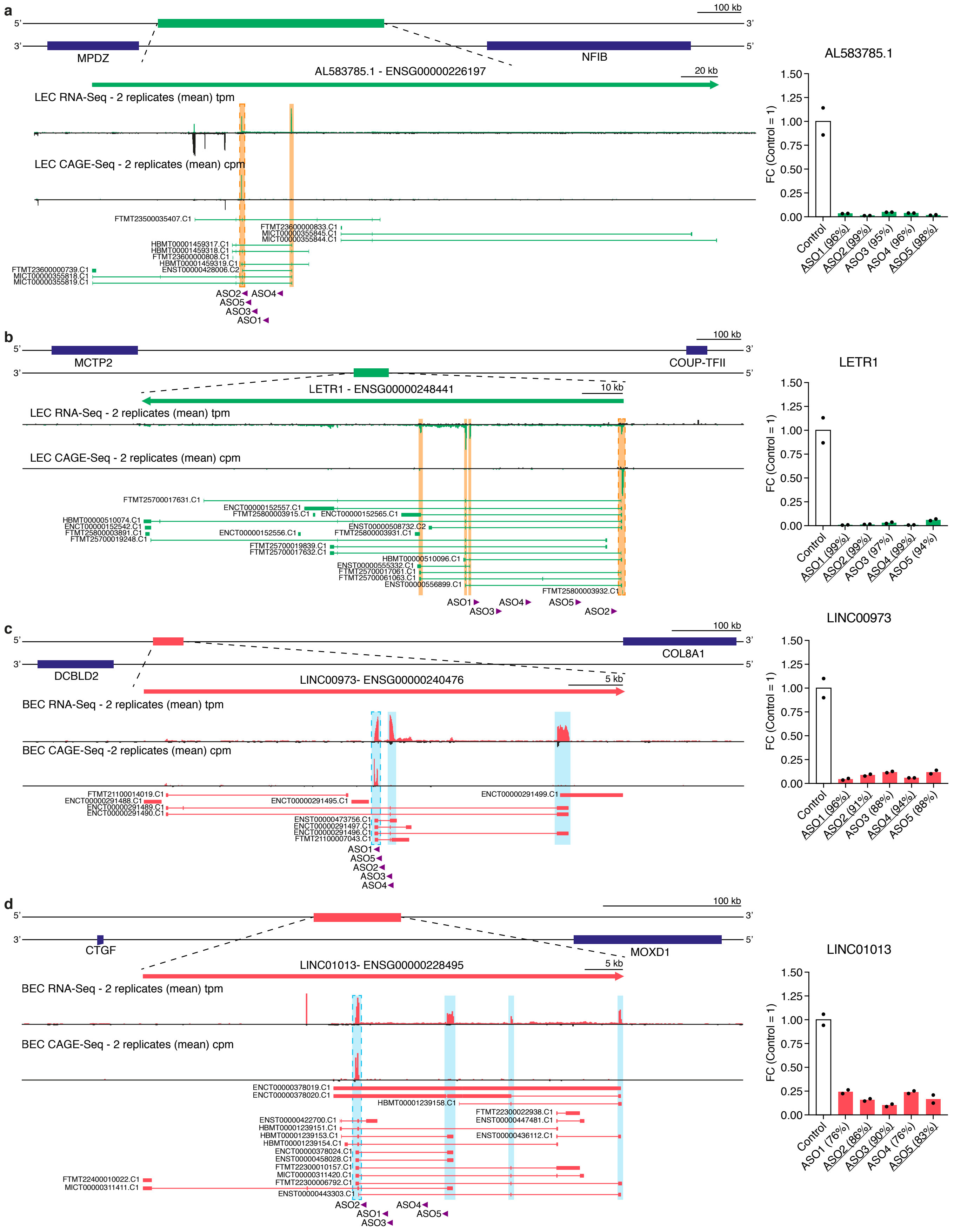

Supplementary Figure 2: ASO design strategy for LEC and BEC lncRNA candidates and selection of 3 most efficient ASOs per target through qPCR.

(a-d) Schematic representation of the genomic regions of 2 LEC (a, b) and 2 BEC (c, d) lncRNA candidates with their flanking genes (blue boxes) according to FANTOM CAT database[3]. Magnifications show lncRNA gene region with respective RNA-Seq (TPM, 2 replicates) and CAGE-Seq (CPM, 2 replicates) signals in LECs (in green) or BECs (in red), related transcripts (green: LEC; red: BEC), and ASO locations (in purple). RNA-Seq and CAGE-Seq signals were visualized through the Zenbu genome browser[4]. Orange/cyan-dashed boxes: overlap between RNA-Seq and CAGE-Seq peaks. Bar charts show knockdown efficiencies determined by qPCR after 48h transfection of five ASOs targeting the 2 LEC and the 2 BEC lncRNAs in neonatal LECs or BECs derived from the same donor. Selected ASOs for CAGE-Seq are underlined. Bars represent FC compared to scrambled control ASO (FC = 1) as mean values (n = 2). RPLP0 was used as the housekeeping gene. Percentages of knockdown efficiencies for each ASO are shown in brackets. ASO sequences are listed in Supplementary Data 3.

Supplementary Figure 3

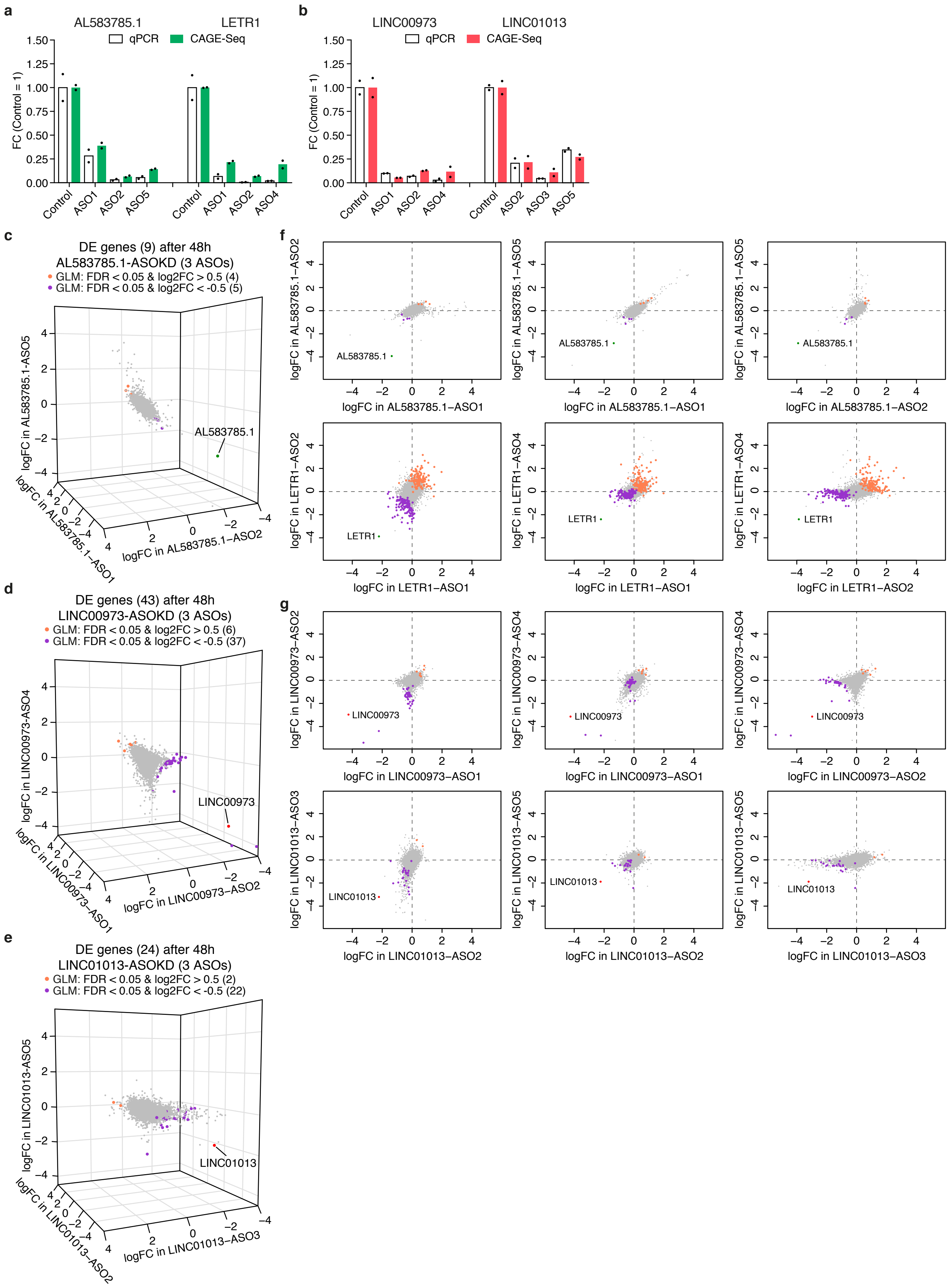

Supplementary Figure 3: Knockdown efficiency of CAGE-Seq samples, DE analysis after ASOKD of AL583785.1, LETR1, LINC00973, and LINC01013, and gating strategy used to sort LECs and BECs from human skin.

(a, b) Graph showing FC reduction compared to scrambled control ASO of LEC (a) and BEC (b) lncRNAs after LETR1-ASOKD determined with qPCR (black bars) and CAGE-Seq (green or red bars). Bars represent mean + SD (n = 2).

(c-e) 3-dimensional scatter plots showing log2FC values calculated between single ASO and scrambled control ASO through EdgeR[5] for LEC candidate AL583785.1 (c) and BEC candidates LINC00973 (d) and LINC01013 (e). Orange and purple dots: significantly (FDR < 0.05) up- and downregulated genes (|log2FC| > 0.5) after differential expression analysis applying a generalized linear model design (GLM)[5]; green dot: AL583785.1; red dots: LINC00973 and LINC01013. DE genes for all lncRNA candidates are listed in Supplementary Data 4.

(f, g) 2-dimensional scatter plots showing log2FC values of ASO pairs for each of the 2 LEC (f) and 2 BEC (g) lncRNAs. Orange and purple dots: up- and downregulated genes; green dots: AL583785.1 and LETR1; red dots: LINC00973 and LINC01013.

Supplementary Figure 4

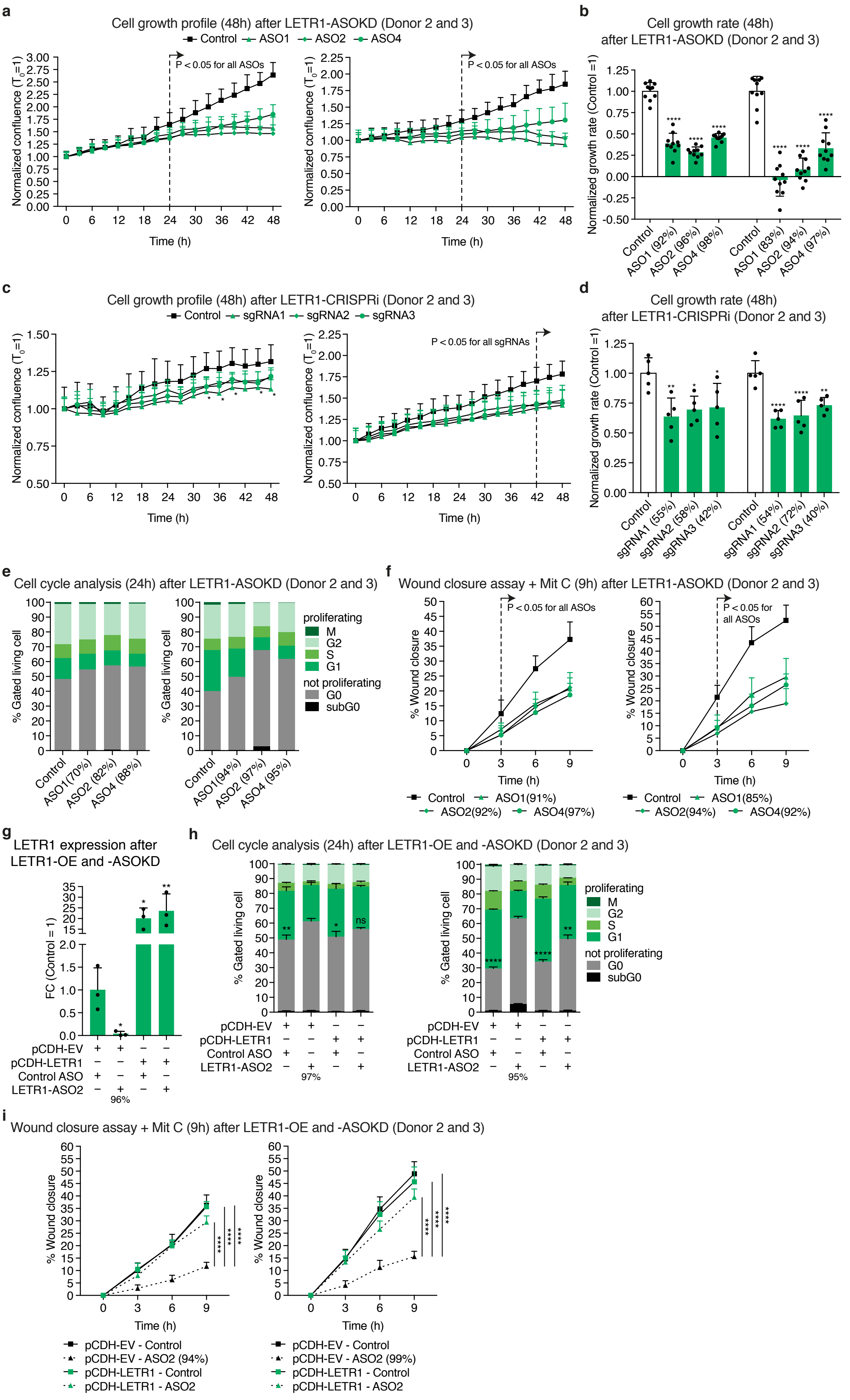

Supplementary Figure 4: Validation of in vitro cell cycle and migration assays in 2 additional donors of LECs.

(a-d) Cell growth profiles and normalized growth rates of LECs over 48h after ASOKD (a, b) or CRIPSRi-KD (c, d) of LETR1.

(e) Quantification of the cell cycle progression analysis of LECs after 24h LETR1-ASOKD.

(f) Quantification of the wound closure assay (up to 9h) of LECs after LETR1-ASOKD.

(g) Expression levels of LETR1 in pCDH-empty vector (pCDH-EV) and pCDH-LETR1 infected neonatal LECs derived from 3 donors after 24h LETR1-ASO2 knockdown. Bars represent FC values against scrambled control ASO. GAPDH was used as the housekeeping genes.

(h) Quantification of the cell cycle progression analysis of pCDH-EV and pCDH-LETR1 infected LECs after 24h LETR1-ASO2 knockdown.

(i) Quantification of the wound closure assay (up to 9h) of pCDH-EV and pCDH-LETR1 infected LECs after LETR1-ASO2 knockdown.

Data are displayed as mean + SD (n = 10 in a, b, f, and i; n = 5 in c and d; n = 3 in g and h; n = 2 in e). Percentages represent LETR1 knockdown efficiencies after the experiments. *P < 0.05, **P < 0.01, ***P < 0.001, ****P < 0.0001, ns: not significant using one-way (for b, d, and h), RM one-way (for g), and two-way (for a, c, f, and i) ANOVA with Dunnett's multiple comparisons test against scrambled control ASO/sgRNA, pCDH-EV – Scrambled control ASO, or LETR1-ASO2 – control siRNA. All displayed in vitro assays were performed in neonatal LECs derived from 2 additional donors.

Supplementary Figure 5

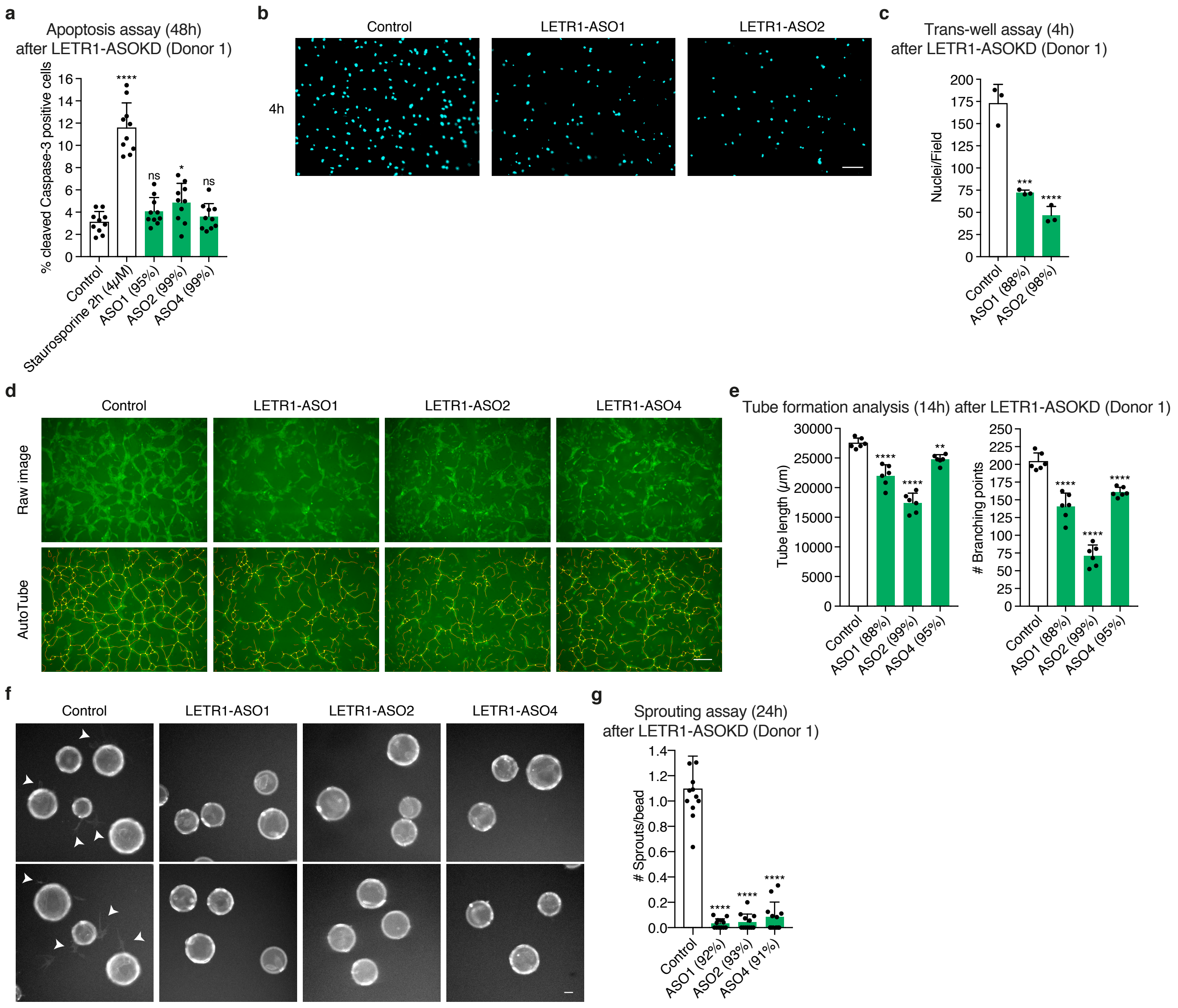

Supplementary Figure 5: Additional in vitro functional studies after LETR1-ASOKD.

(a) Quantification of the apoptosis assay (48h) of LECs after LETR1-ASOKD. Percentages of cleaved caspase 3-positive cells were determined using ImageJ[6]. Cells incubated for 2h with Staurosporine (4μM) were used as a positive control.

(b) Representative images of the trans-well assay (4h) of LECs after LETR1-ASOKD. Nuclei were stained with DAPI. Scale bar represents 120μm.

(c) Quantification of the trans-well assay (4h) of LECs after LETR1-ASOKD. DAPI-stained nuclei per field were determined using ImageJ[6].

(d) Representative images of the tube formation assay (14h) of LECs after LETR1-ASOKD. LECs were pre-stained with CellTracker green to allow automated quantification using AutoTube[7]. Scale bar represents 200μm.

(e) Quantification of the tube formation assay (14h) of LECs after LETR1-ASOKD. Tube length and branching point were calculated using AutoTube[7].

(f) Representative images of the sprouting assay (24h) of LECs after LETR1-ASOKD. LECs were stained for Phalloidin to allow manual counting of the sprouted cells. Scale bar represents 200μm.

(**g**) Quantification of the sprouting assay (24h) of LECs after LETR1-ASOKD. Sprouted cells were counted manually using ImageJ[6]. White arrows indicate LEC sprout.

Data are displayed as mean + SD (n = 12 in g; n = 10 in a; n = 6 in e; n = 3 in c). Percentages represent LETR1 knockdown efficiencies after the experiments. *P < 0.05, **P < 0.01, ***P < 0.001, ****P < 0.0001, ns: not significant using one-way ANOVA with Dunnett's multiple comparisons test against scrambled control ASO. All displayed in vitro assays were performed in neonatal LECs derived from the same donor.

Supplementary Figure 6

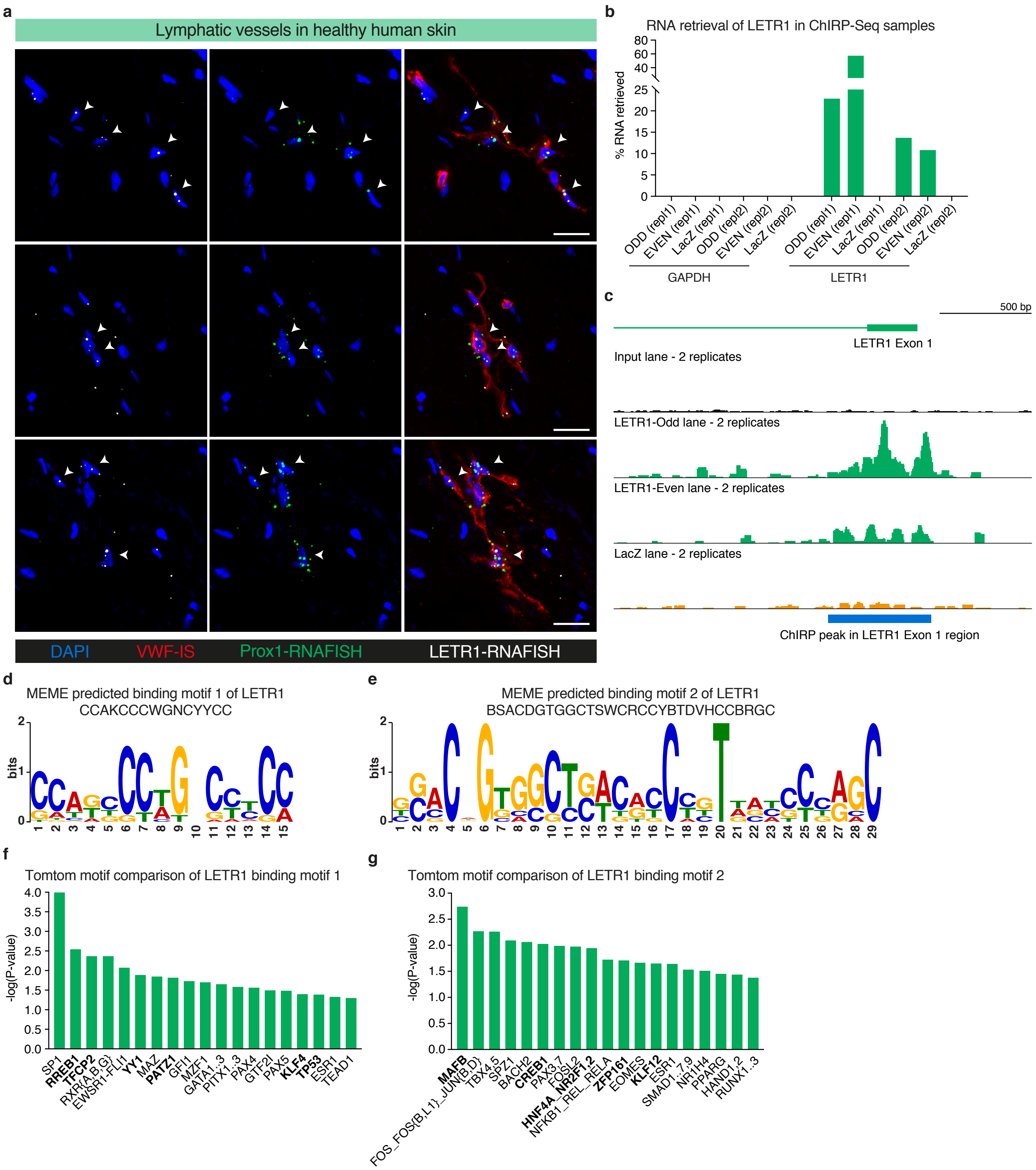

Supplementary Figure 6: LETR1 in vivo expression of LETR1 and results after LETR1 ChIRP-Seq.

(a) Representative images of lymphatic vessels in healthy human skin samples derived from 2 donors. Lymphatic vessels were defined as von Willebrand factor (vWF, red – immunostaining) and PROX1 (green – smRNA-FISH) double positive. Scale bars represent 20μm.

(b) RNA retrieval displayed as percentages of GAPDH (negative control) and LETR1 in ODD, EVEN, and LacZ samples (2 replicates of neonatal LECs derived from the same donor). Probe sequences are listed in Supplementary Data 9.

(c) Schematic representation of the genomic region of LETR1 – Exon 1 and the corresponding ChIRP-Seq signal of input (black), LETR1-Odd and -Even (green), and LacZ lanes (orange). Significant peak region is shown in blue. ChIRP-Seq signals were visualized through Integrative Genomics Viewer (IGV)[8].

(d, e) Predicted LETR1 motifs found in 19 out of the 53 binding sites present in the 44 final target gene bodies as determined by MEME[9] (E-value: $2.01x10^{-6}$ – motif 1; E-value: $7.90x10^{-6}$ – motif 2).

(f, g) Transcription factor motifs similar to the predicted LETR1 binding motifs, as determined by Tomtom[10]. Overlapping transcription factors with the MARA results (Figure 3e, f) are displayed in bold.

Supplementary Figure 7

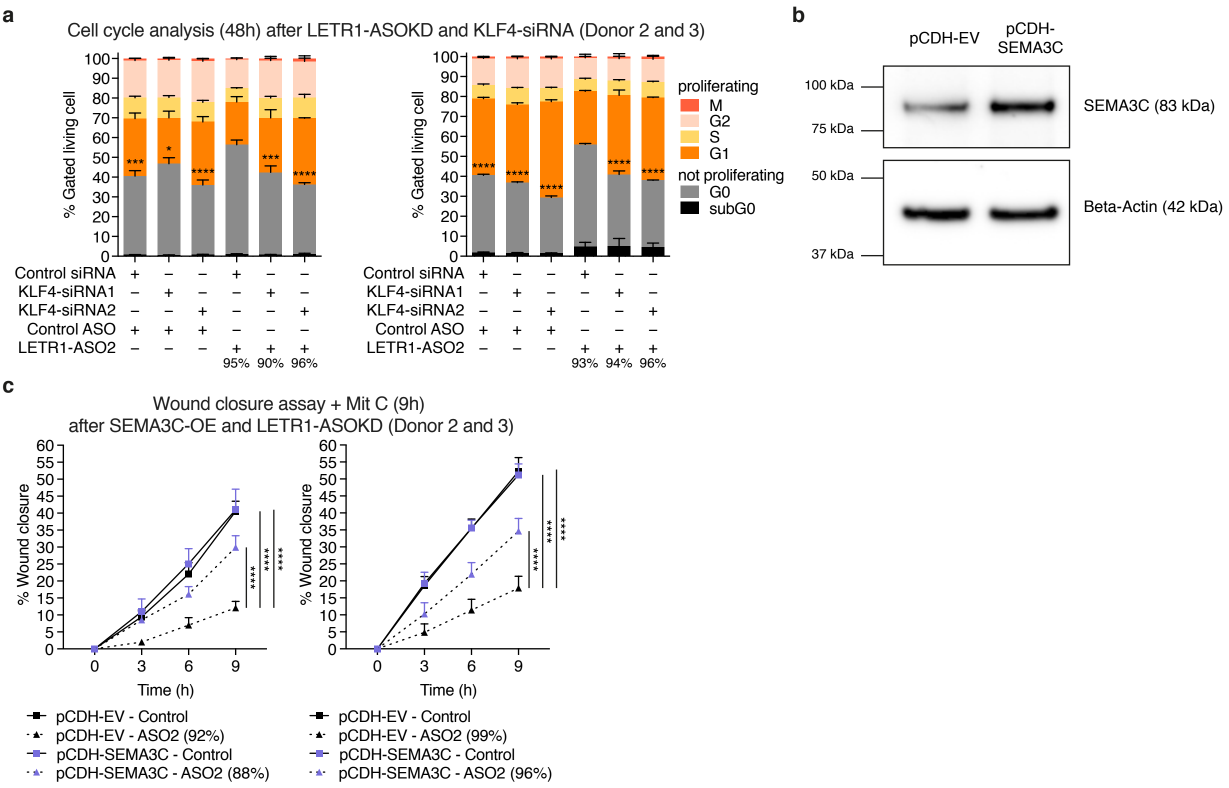

Supplementary Figure 7: Validation of KLF4 and SEMA3C rescues in 2 additional donors of LECs.

(a) Quantification of the cell cycle progression analysis of LECs after 24h LETR1-ASO2 knockdown followed by 24h siRNA-KD of KLF4.

(b) Western blot images for SEMA3C in pCDH-EV and pCDH-SEMA3C. Uncropped western blot image is shown in Supplementary Figure 9.

(c) Quantification of the wound closure assay (up to 9h) of pCDH-EV and pCDH-SEMA3C infected LECs after LETR1-ASO2 knockdown.

Data are displayed as mean + SD (n = 3 in a; n = 10 in c). Percentages represent the knockdown efficiencies of LETR1 after the experiments. *P < 0.05, ***P < 0.001, ****P < 0.0001 using, ordinary one-way (for a), and two-way (for c) ANOVA with Dunnett's multiple comparisons test against LETR1-ASO2 – control siRNA or pCDH-EV – ASO2. All displayed in vitro assays were performed in neonatal LECs derived from 2 additional donors.

Supplementary Figure 8

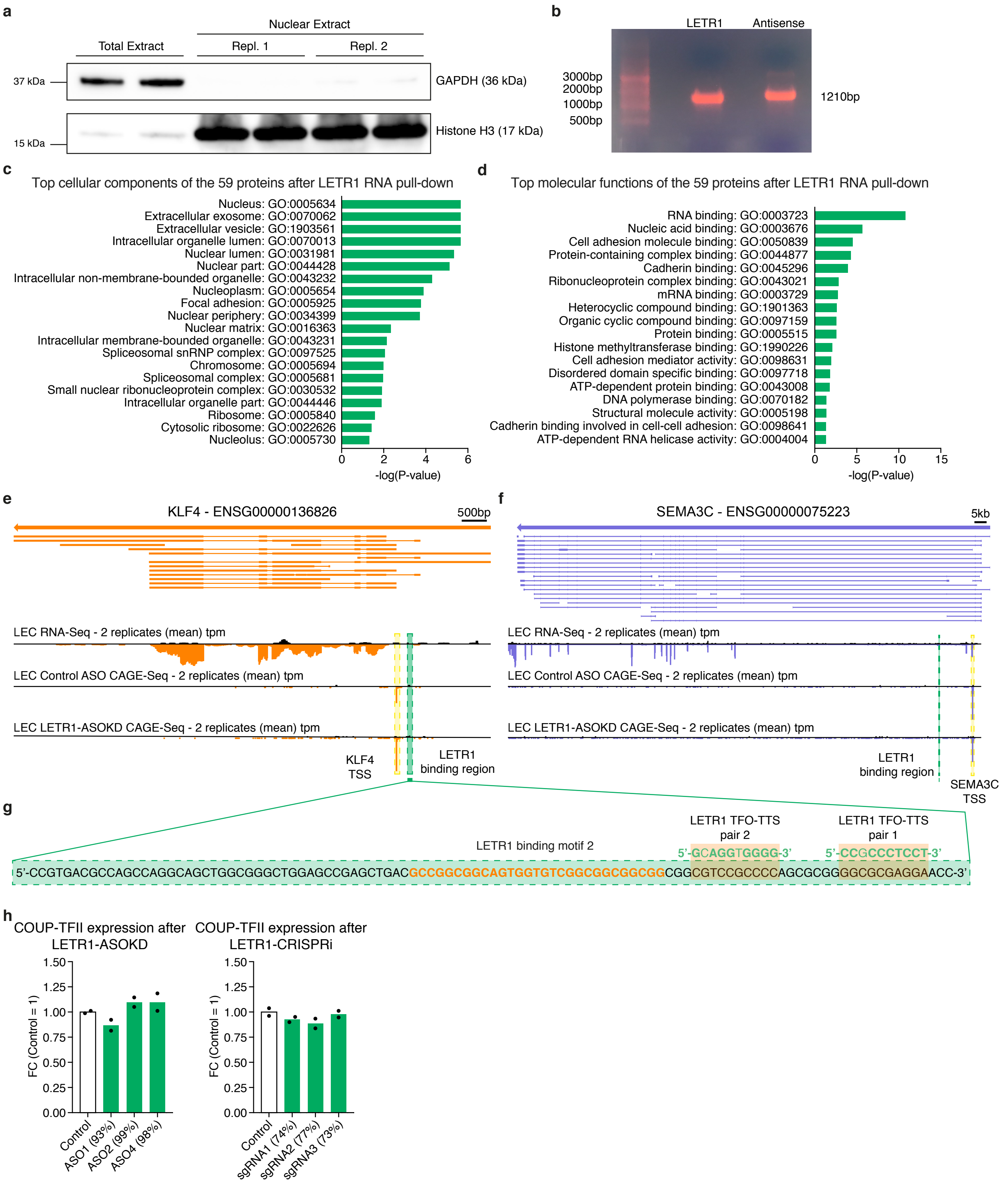

Supplementary Figure 8: LETR1 RNA pull-down results.

(a) Evaluation of the subcellular fractionation of nuclear compared to total extracts from neonatal LECs after performing western blot of GAPDH (cytoplasmic protein) and Histone H3 (nuclear protein). Uncropped western blot image is shown in Supplementary Figure 9.

(b) Gel electrophoresis showing the fragment size of biotin-LETR1 and antisense biotin-RNA control.

(c, d) Top significantly (P-value < 0.05) enriched GO terms for cellular components (c) and molecular functions (d) of the 59 proteins after LETR1 RNA pull-down, using g:ProfileR[2] (relative depth 4-8 for (c) and 1-4 for (d)).

(e, f) Schematic representation of the genomic regions of KLF4 (e, orange) and SEMA3C (f, purple) with related transcripts and RNA-Seq (TPM, 2 replicates) and CAGE-Seq LETR1-ASOKD (CPM, 2 replicates) signals in LECs. RNA-Seq and CAGE-Seq signals were visualized through the Zenbu genome browser[4]. Yellow-dashed boxes: gene target TSSs; green-dashed boxes: LETR1 binding site regions.

(g) Schematic representation of LETR1 binding region in the KLF4 gene body with the location of the predicted LETR1 binding motif 2 and the two TFO-TSS pairs identified via MEME[9] and Triplexator[11], respectively

(h) Expression quantification of COUP-TFII after LETR1-ASOKD and LETR1-CRISPRi followed by qPCR. Percentages display the knockdown efficiency of LETR1 after the experiments. Data are represented as mean values (n = 2).

Supplementary Figure 9

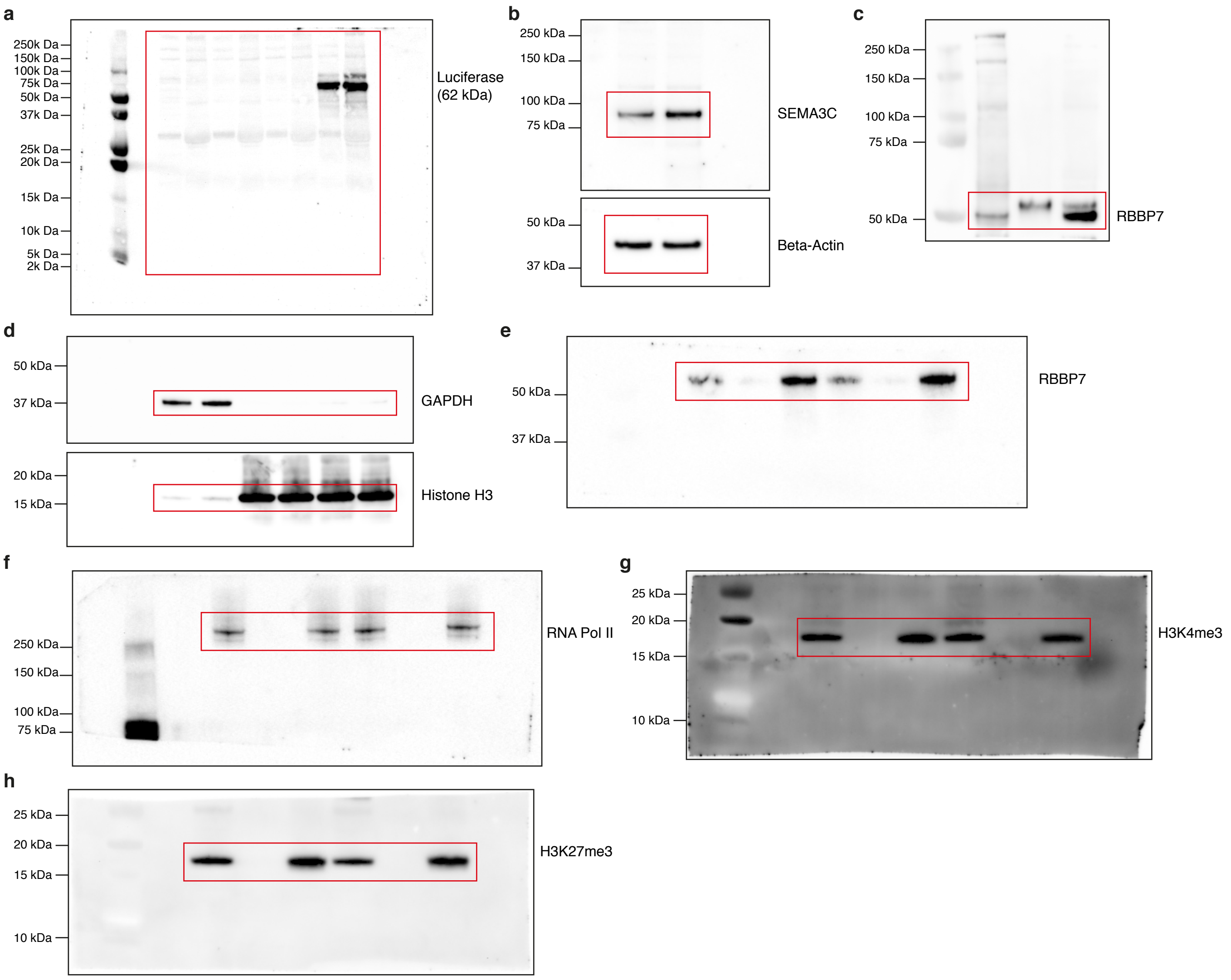

Supplementary Figure 9: Uncropped western blot images.

(a) Western blot images for the in vitro translation assay from Figure 4e.

(b) Western blot images for SEMA3C from Supplementary Figure 7b.

(c) Western blot image for RBBP7 from Figure 8c.

(d) Western blot image for GAPDH and Histone H3 from Supplementary Figure 8a.

(e-h) Western blot image for RBBP7 (e), RNA Pol II (f), H3k4me3 (g), and H3K27me3 (h) after ChIP-qPCR from Figure 8f.

In all panels, red boxes represent the cropped region displayed in the corresponding figure.

References Supplementary Information

1. McLean, C. Y. *et al.* GREAT improves functional interpretation of cis-regulatory regions. *Nat. Biotechnol.* **28,** 495–501 (2010).
2. Reimand, J., Kull, M., Peterson, H., Hansen, J. & Vilo, J. g:Profiler--a web-based toolset for functional profiling of gene lists from large-scale experiments. *Nucleic Acids Res.* **35,** W193–200 (2007).
3. Hon, C.-C. *et al.* An atlas of human long non-coding RNAs with accurate 5' ends. *Nature* **543,** 199–204 (2017).
4. Severin, J. *et al.* Interactive visualization and analysis of large-scale sequencing datasets using ZENBU. *Nat. Biotechnol.* **32,** 217–219 (2014).
5. Robinson, M. D., McCarthy, D. J. & Smyth, G. K. edgeR: a Bioconductor package for differential expression analysis of digital gene expression data. *Bioinformatics* **26,** 139–140 (2010).
6. Schindelin, J. *et al.* Fiji: an open-source platform for biological-image analysis. *Nat. Methods* **9,** 676–682 (2012).
7. Montoya-Zegarra, J. A. *et al.* AutoTube: a novel software for the automated morphometric analysis of vascular networks in tissues. *Angiogenesis* **22,** 223–236 (2019).
8. Robinson, J. T. *et al.* Integrative genomics viewer. *Nat. Biotechnol.* **29,** 24–26 (2011).
9. Bailey, T. L. & Elkan, C. Fitting a mixture model by expectation maximization to discover motifs in biopolymers. *Proc Int Conf Intell Syst Mol Biol* **2,** 28–36 (1994).
10. Gupta, S., Stamatoyannopoulos, J. A., Bailey, T. L. & Noble, W. S. Quantifying similarity between motifs. *Genome Biol.* **8,** R24–9 (2007).
11. Buske, F. A., Bauer, D. C., Mattick, J. S. & Bailey, T. L. Triplexator: detecting nucleic acid triple helices in genomic and transcriptomic data. *Genome Research* **22,** 1372–1381 (2012).
