# Supplementary material for: LETR1 is a lymphatic endothelial-specific lncRNA governing cell proliferation and migration through KLF4 and SEMA3C
Source: Nat Commun. 2021 Feb 10;12:925. doi: 10.1038/s41467-021-21217-0 (PMC7876020; doi:10.1038/s41467-021-21217-0)
Supplement: Supplementary file 16 — Reporting Summary [file 41467_2021_21217_MOESM16_ESM.pdf]

nature research

Corresponding author(s): Prof. Dr. Michael Detmar

Last updated by author(s): 2021/01/17

Reporting Summary

Nature Research wishes to improve the reproducibility of the work that we publish. This form provides structure for consistency and transparency in reporting. For further information on Nature Research policies, see Authors & Referees and the Editorial Policy Checklist .

Statistics

For all statistical analyses, confirm that the following items are present in the figure legend, table legend, main text, or Methods section.

n/a	Confirmed	
☐	☒	The exact sample size (*n*) for each experimental group/condition, given as a discrete number and unit of measurement
☐	☒	A statement on whether measurements were taken from distinct samples or whether the same sample was measured repeatedly
☐	☒	The statistical test(s) used AND whether they are one- or two-sided<br>*Only common tests should be described solely by name; describe more complex techniques in the Methods section.*
☒	☐	A description of all covariates tested
☐	☒	A description of any assumptions or corrections, such as tests of normality and adjustment for multiple comparisons
☐	☒	A full description of the statistical parameters including central tendency (e.g. means) or other basic estimates (e.g. regression coefficient) AND variation (e.g. standard deviation) or associated estimates of uncertainty (e.g. confidence intervals)
☐	☒	For null hypothesis testing, the test statistic (e.g. *F*, *t*, *r*) with confidence intervals, effect sizes, degrees of freedom and *P* value noted<br>*Give P values as exact values whenever suitable.*
☒	☐	For Bayesian analysis, information on the choice of priors and Markov chain Monte Carlo settings
☒	☐	For hierarchical and complex designs, identification of the appropriate level for tests and full reporting of outcomes
☐	☒	Estimates of effect sizes (e.g. Cohen's *d*, Pearson's *r*), indicating how they were calculated

Our web collection on statistics for biologists contains articles on many of the points above.

Software and code

Policy information about availability of computer code

Data collection	FACSDiva (ver. 6.1.3), BD Biosciences; IncuCyte ZOOM (ver. 2016B), Essen Bioscience; Mascot (ver. 2.5.1.3), Matrix Sciences.
Data analysis	Scaffold (ver. 4.8.8), Proteome Software; GraphPad Prism (ver. 7.0.0), Graphpad; CLC Genomic Workbench (ver. 10.1.1), Qiagen; FlowJo (ver. 10.lr3), BD Biosciences; Image J (ver. 2.0.0-rc-69/1.52i), Schindelin et al., 2012; TScratch, Gebäck et al., 2009. EdgeR (ver. 3.12.1), Robinson et al., 2010; McCarthy et al., 2012; SuperExactTest (ver. 1.0.0), M. Wang et al., 2015; GREAT web tool (ver. 4.0.4), McLean et al., 2010; g:Profiler (ver 0.6.7), Reimand et al., 2007; GSEA analysis xtools.gsea.Gsea from javascript gsea2-2.2.4.jar, Mootha et al., 2003; Subramanian et al. 2005; MARA analysis, Alam et al., 2020; SolexaQA (ver. 3.1.7.1), Cox et al., 2010; Bowtie (ver. 1.1.1), Langmead et al., 2009; MACS 2.0 (ver. 2.1.1), Y. Zhang et al., 2008; Bedtools (ver. 2.27.1), Quinlan and Hall, 2010; Circos (ver. 0.69-7), Krzywinski et al., 2009; STRING web tool, Szklarczyk et al., 2019; Cytoscape (ver. 3.6.1) with Enrichment Map and Wordcloud plugin, Shannon et al., 2003; AutoTube (ver. 1.0), Montoya-Zegarra et al., 2019; MEME (ver. 5.1.1), Bailey and Elkan, 1994; Tomtom (ver. 5.1.1), Gupta et al., 2007; Triplexator, Buske et al., 2012; VirtualBox software (ver. 6.1); ViennaRNA package (ver. 2.0), Lorenz et al., 2011.

For manuscripts utilizing custom algorithms or software that are central to the research but not yet described in published literature, software must be made available to editors/reviewers. We strongly encourage code deposition in a community repository (e.g. GitHub). See the Nature Research guidelines for submitting code & software for further information.

Data

Policy information about availability of data

All manuscripts must include a data availability statement. This statement should provide the following information, where applicable:
- Accession codes, unique identifiers, or web links for publicly available datasets
- A list of figures that have associated raw data
- A description of any restrictions on data availability

All unprocessed sequencing data are deposited in the DDBJ DRA public repository with the following accession numbers: DRA009940 (CAGE-Seq, https://www.ncbi.nlm.nih.gov/sra/?term=DRA009940), DRA009941 (ChIRP-Seq, https://www.ncbi.nlm.nih.gov/sra/?term=DRA009941), and DRA009942 (RNA-Seq, https://

www.ncbi.nlm.nih.gov/sra/?term=DRA009942). LC/MS data are available at the ProteomXchange (via PRIDE) with the following accession number: PXD018578 (https://www.ebi.ac.uk/pride/archive/projects/PXD018578). Databases used in the study: FANTOM CAT (https://fantom.gsc.riken.jp/cat/v1/#/), FANTOM6 (https://fantom.gsc.riken.jp/6/), human PPI (via STRING, https://string-db.org/cgi/input.pl), human and mouse SwissRegulon (via MEME suite, http://meme-suite.org/), g:Profiler Ensembl 90, Ensembl Genome 37 (https://biit.cs.ut.ee/gprofiler_archive3/r1741_e90_eg37/web/), swissprot homo sapiens proteome (via Mascot software). All data are available from the corresponding author, Dr. Michael Detmar (michael.detmar@pharma.ethz.ch), upon reasonable request. Source data are provided with this paper.

Field-specific reporting

Please select the one below that is the best fit for your research. If you are not sure, read the appropriate sections before making your selection.

[x] Life sciences [ ] Behavioural & social sciences [ ] Ecological, evolutionary & environmental sciences

For a reference copy of the document with all sections, see nature.com/documents/nr-reporting-summary-flat.pdf

Life sciences study design

All studies must disclose on these points even when the disclosure is negative.

Sample size	No specific methods were used for sample size determination. Sample size for all the presented experiments was determined to be at least two (n = 2) to provide sufficient data for downstream analysis.
Data exclusions	No data were excluded
Replication	As indicated in the figure legends, all experiments presented in this study were successfully replicated at least in duplicates either using cells deriving from the same donor or from different donors.
Randomization	No randomization was necessary since all the in vitro experiments presented in this study were at least duplicated to confirm the results. All samples were treated side-by-side under the same conditions and compared to relevant controls.
Blinding	No blinding was necessary since all the in vitro experiments presented in this study were at least duplicated to confirm the results.

Reporting for specific materials, systems and methods

We require information from authors about some types of materials, experimental systems and methods used in many studies. Here, indicate whether each material, system or method listed is relevant to your study. If you are not sure if a list item applies to your research, read the appropriate section before selecting a response.

Materials & experimental systems

n/a	Involved in the study	
[ ]	[x]	Antibodies
[ ]	[x]	Eukaryotic cell lines
[x]	[ ]	Palaeontology
[x]	[ ]	Animals and other organisms
[ ]	[x]	Human research participants
[x]	[ ]	Clinical data

Methods

n/a	Involved in the study	
[x]	[ ]	ChIP-seq
[ ]	[x]	Flow cytometry
[x]	[ ]	MRI-based neuroimaging

Antibodies

Antibodies used	Alexa647-conjugated mouse anti-human podoplanin antibody, Novus Biologicals, Clone 18H5; Cat#: NB600-1013AF647 ; PE-conjugated mouse anti-human CD31 antibody, BD Pharmingen, Clone WM59; Cat#: 550389; Mouse anti-human Ki-67 antibody, Dako, Cat#: M7240; Mouse IgG isotype control, R&D Systems, Clone 11711, Cat#: MAB002; Mouse anti-human CD34 biotinylated antibody, Thermo Fisher Scientific, clone 581; Cat#: CD34-581-15; Secondary goat anti-rabbit IgG antibody, Dako, Cat#: P0448; Secondary rabbit anti-goat IgG antibody, R&D Systems, Cat#: HF017; Donkey Alexa488-conjugated anti-mouse secondary antibody, Thermo Fisher Scientific, Cat#: A21202; Rabbit anti-cleaved caspase 3 (Asp175) antibody, Cell Signaling, Cat#: 9661; donkey Alexa488-conjugated anti-rabbit secondary antibody, Thermo Fisher Scientific, Cat#: A21206; Mouse anti-human CD31 antibody, Dako, Clone JC70A; Cat#: M0823; Rabbit anti-human vWF antibody, Dako, Cat#: A0082; FITC-conjugated mouse anti-human CD45 antibody, Biolegend, Clone HI30; Cat#: 304006; Rabbit anti-human SEMA3C, Thermo Fisher Scientific, Cat#: PA5-67973, Rabbit anti-beta-actin antibody, Abcam, Cat#: ab8227; eFluor 450-conjugated rat anti-human Ki-67 antibody, ebioscience, Clone: SolA15; Cat#: 48-5698-80, rabbit anti-human GAPDH antibody, Sigma, Cat#: G9545; rabbit anti-histone H3 antibody, Sigma, Cat#: H1064; rabbit anti-human RBBP7 antibody, Cell Signaling, Cat#: 6882; mouse anti-rabbit IgG conformation-specific secondary antibody, Cell Signaling, Cat#: 3678; Rabbit IgG isotype control antibody, Sigma, Cat#: I5006; Rabbit anti-human H3K27me3 antibody, Diagenode, Cat#: C15410195; Rabbit anti-human H3K4me3 antibody, Diagenode, Cat#: C15410003-50; Mouse anti-human RNA Pol II antibody, Sigma, Cat#: 17-620; Mouse IgG isotype control antibody, Sigma, Cat#: 17-620; Goat HRP-conjugated secondary anti-mouse IgG antibody, Dako, Cat#: P0447; Mouse anti-human RBBP7 antibody, Origene, Clone: OTI3A12 Cat#: TA503809.
Validation	All antibodies were used according to the manufacturers' recommendations. All the companies provide quality certificates for the antibodies used in the study. Each antibody was first tested with different dilutions, and optimal dilution ratios used in the study are stated in the methods section.

Eukaryotic cell lines

Policy information about cell lines

Cell line source(s)	Lymphatic and blood vascular endothelial cells (LECs and BECs) from the Detmar Lab.<br>HEK293T cells were kindly provided by the Christian Munz Lab (University of Zurich).
Authentication	Cell line used were not authenticated.
Mycoplasma contamination	Cells were routinely tested for mycoplasma, and we confirm that every cell used in this study was found negative.
Commonly misidentified lines<br>(See ICLAC register)	No commonly misidentified cell lines were used.

Human research participants

Policy information about studies involving human research participants

Population characteristics	Adult samples were collected from the following five patients:<br>- female, 37 years old, location: abdomen or thigh, condition: dermatochalasis, acne inversa, obese, antibiotics: Clindamycin rifampicin<br>- female, 51 years old, location: breast, condition: breast reduction, no syndromes<br>- male, 72 years old, location: breast, condition: no syndromes<br>- female, 45 years old, location: breast, condition: breast reduction, no syndromes<br>- female, 47 years old, location: abdomen, condition: breast cancer<br><br>Neonatal samples were obtained from three donors after routine circumcisions.
Recruitment	Adult samples were collected after obtaining written informed consensus from adult subjects (see above) admitted for plastic surgery at the University Hospital Zurich.<br><br>Neonatal samples were collected after obtaining written informed consensus from the parents at the Massachusetts General Hospital, Boston, MA.
Ethics oversight	For adult samples: Ethics Committee of the Kanton Zurich (2017-00687).<br>For neonatal samples: Human Research Committee of the Massachusetts General Hospital, Boston, MA (IRB protocol number 1999-P-009609/5).

Note that full information on the approval of the study protocol must also be provided in the manuscript.

Flow Cytometry

Plots

Confirm that:

- [x] The axis labels state the marker and fluorochrome used (e.g. CD4-FITC).
- [x] The axis scales are clearly visible. Include numbers along axes only for bottom left plot of group (a 'group' is an analysis of identical markers).
- [x] All plots are contour plots with outliers or pseudocolor plots.
- [x] A numerical value for number of cells or percentage (with statistics) is provided.

Methodology

Sample preparation

For sorting of LECs and BECs: ECs were obtained from the abdominal or breast skin of healthy adult subjects admitted for plastic surgery at the University Hospital Zurich. Written informed consent was obtained from each donor/tissue collection, as approved by the Ethics Committee of the Kanton Zurich (2017-00687). Skin samples were washed in Hank's balanced salt solution (HBSS) supplemented with 5% fetal bovine serum (FBS, Gibco), 2% antibiotic and antimycotic solution (AA, Gibco) and 20mM HEPES (Gibco), and subsequently incubated in 0.25% trypsin (Sigma) diluted in DPBS (Gibco) with the dermal side facing downwards overnight at 4°C. Trypsin digestion was stopped by washing the tissues with RPMI basal medium supplemented with 10% FBS, 2% AA, and 20mM HEPES. After removal of the epidermal sheets, the dermis was finely minced and enzymatically digested (RPMI basal medium, 1000U/mL collagenase type 1 (Worthington), 40µg/mL DNase I (Roche)) for 1h at 37°C under constant agitation. Digested tissues were then filtered through a 100µm cell strainer (Falcon), washed with RPMI basal medium, and centrifuged at 1,500rpm for 6min at 4°C.

For analysis of LEC- and BEC-specific lncRNAs in adult LECs and BECs: Cells were seeded into fibronectin (Roche) coated plates and were cultured in EGM-2-MV complete medium (Lonza). After 7-10 days, cells were trypsinized and endothelial cells were selected based on CD31 positivity with Dynabeads CD31 endothelial cell magnetic beads (Thermo Fisher Scientific) and cultured until confluency. Endothelial cells were detached, washed with FACS buffer (DPBS with 2% FBS and 1mM EDTA), and stained with Alexa647-conjugated mouse anti-human podoplanin antibody (1:70, clone 18H5, Novus Biologicals) and PE-conjugated mouse anti-human CD31 antibody (1:20, clone WM59, BD Pharmingen) in FACS buffer for 30min at 4°C. After a wash with FACS buffer, endothelial cells were finally sorted on a FACSAria II (BD Biosciences) with a 70µm nozzle, using FACSDiva software.

For ex-vivo analysis of LETR1 expression: Subsequently, isolated single cells were stained with mouse anti-human CD34 biotinylated antibody (clone 581, Thermo Fisher Scientific) diluted 5µL for 1 million cells in FACS buffer (DPBS with 2% FBS and 1mM EDTA) for 30min at 4°C. After washing once with FACS buffer, isolated single cells were co-stained in FACS buffer with FITC-conjugated mouse anti-human CD45 antibody (1:25; clone HI30, Biolegend), PE-conjugated mouse anti-human CD31 antibody (1:25; clone WM59, BD Pharmingen), PerCP-conjugated streptavidin (1:400; Biolegend), Zombie NIR (1:500; BioLegend) for 30min at 4°C. After washing in FACS buffer, isolated single cells were filtered and sorted for living, CD45-CD31+ CD34low (LEC), or CD34 high (BEC) directly into test tubes containing 250µL RLT plus lysis buffer, using a FACSAria (BD Biosciences).

Instrument

FACSAria II (BD Biosciences)

Software

FlowJo (BD Biosciences)

Cell population abundance

Lymphatic and blood vascular endothelial cells were sorted based on the gating strategy below and purity of both cells was determined by FMO controls and qPCR of lineage-specific markers

Gating strategy

lymphatic endothelial cells: CD45-; CD31+; CD34-
blood vascular endothelial cells: CD45-; CD31+; CD34+

- [x] Tick this box to confirm that a figure exemplifying the gating strategy is provided in the Supplementary Information.
